# Prevalence of domestic violence in a time of catastrophic disease outbreaks including COVID-19 pandemic: a systematic review protocol

**DOI:** 10.1186/s13643-022-01920-9

**Published:** 2022-03-17

**Authors:** Gelila Abraham, Beshea Gelana, Kiddus Yitbarek, Sudhakar Morankar

**Affiliations:** 1 Department of Health Policy and Management, Public Health Faculty, Institute of Health, Jimma University, Jimma, Ethiopia; 2 Ethiopian Evidence-Based Healthcare Centre, Health Behaviour and Society Department, Public Health Faculty, Institute of Health, Jimma University, Jimma, Ethiopia; 3 Department of Health Behaviour and Society, Public Health Faculty, Institute of Health, Jimma University, Jimma, Ethiopia

**Keywords:** Domestic violence, Disease outbreak, Pandemic, Epidemic, Prevalence, Vulnerable groups

## Abstract

**Background:**

Domestic violence is a public health issue that has a long-term and irreversible effect on the victims. There are vulnerable groups like children, women, and elders. The problem becomes worse for these populations in the time of catastrophic events including disease pandemics. However, few attempts have been made to systematically review the prevalence and pattern of domestic violence during these times all over the world.

**Methods:**

An initial search of PubMed will be followed by CINAHL, Scopus, Google Scholar, Embase, and ProQuest Health. The titles and abstracts of studies will be reviewed, and full-text articles will be selected if the inclusion criteria are met. Studies that meet the eligibility criteria will then be assessed by two independent reviewers. Full-text articles will be selected if the inclusion criteria are met. A standardized critical appraisal checklist for studies reporting prevalence data will be used to assess the methodological quality, and a standardized data extraction tool will be used. The results from the included studies will be analysed using the JBI SUMARI software.

**Discussion:**

This systematic review will provide solid evidence on the magnitude of domestic violence of any forms during catastrophic disease outbreaks including the current pandemic, COVID-19.

**Systematic review registration:**

PROSPERO CRD42020192255.

**Supplementary Information:**

The online version contains supplementary material available at 10.1186/s13643-022-01920-9.

## Background

Domestic violence is defined broadly as ‘all acts of physical, sexual, psychological, or economic violence’ that may be committed by a family member or intimate partner [1]. The United Nations name it ‘domestic abuse’ or ‘intimate partner violence’ and defined it as ‘a pattern of behaviour in any relationship that is used to gain or maintain power and control over an intimate partner’, and this violence could be physical, sexual, and emotional (psychological) [2]. Besides, the World Health Organization (WHO) defines violence as ‘the intentional use of power or force, threatened or actual, against oneself, another person or against a group or community, which either results in or has a likelihood of resulting in, injury, death, psychological harm, mal-development, or deprivation’. Based on this definition, WHO separates violence into three broad groups, namely, self-directed violence, interpersonal violence, and collective violence. Self-directed violence includes suicidal behaviour (i.e. suicidal ideation, plans, attempted suicide, and suicide). Interpersonal violence by itself divides into two categories, i.e. family and intimate partner violence (e.g. child abuse, violence by an intimate partner, and abuse of the elderly) and community violence (e.g. youth violence, rape or sexual assault by strangers, and violence in institutional settings). Collective violence includes wars and armed conflicts within or between states, genocide, and terrorism [3].

It is well known that violence has probably always been part of the human experience, and its impact can be seen, in various forms, in all parts of the world. All types of violence are related to human distress and disastrous. Natural disasters including pandemics might increase the rate of violence both in the short and long terms in a number of ways [4]. Domestic violence is the most common form of violence against children, women, and elderly persons. It affects the victims up to the life span, from self-selective to forced suicide and abuse, and is evident, to some degree, in every society in the world. There is a huge body of research showing the lasting effects of domestic violence on victims which includes mental health problems and chronic illnesses [5]. There is also evidence which shows, in 1 year, approximately 300 million women aged 15–64 are assaulted by an intimate partner, that is, every ninth woman in the world, every year [6]. There is increasing recognition internationally that intimate partner violence is a common problem with serious health, social, and economic consequences for women, their families, and communities [7].

Domestic violence is now viewed as global public health and clinical problem of epidemic proportions. For women and girls, being subjected to violence is associated with injury, disability, death, induced abortion, low birth weight and prematurity in women’s babies, poor sexual health, suicide, depression, anxiety, and harmful alcohol use [8]. In the middles of disaster, be it a natural disaster or disease outbreak, the physical and social environments that shape the health and health problems will disrupt. Therefore, the effects of disasters are likely to increase in individuals, families, and communities’ vulnerability to violence and effects can have both an immediate and long-term impact on violence [9]. Furthermore, due to the scarcity of basic provisions, failure of law enforcement, powerlessness, aggravation caused by a deceitful bureaucracy, and forgotten governments’ potentials to help victims, etc., it would be possible that the mental distresses will develop into violence, either self-directed or interpersonal [10]. Likewise, studies recently conducted revealed that interpersonal violence increases during epidemics, like the Ebola virus in West Africa and currently COVID-19 pandemics, globally [11, 12].

There is an evidence showing domestic violence reports often substantially increase after a catastrophic event [13]. While the depth and severity of the COVID-19 pandemic are still uncertain, it is clear that households in affected areas will feel some economic shock—with the largest effects among the population of already economically vulnerable. In line with this, women, in particular, may be disproportionately affected by additional unpaid care (caretaking and caregiving) work, which may further decrease the ability to undertake paid work [14]. Furthermore, there is a high likelihood of increases in family violence during the COVID-19 pandemic [15]. Disruption of family life has a significant effect on children, including poverty (if divorce or separation occurs) and a loss of faith and trust in the association of the family. These squeals not only affect the quality of lives of individuals and societies but also have lasting effects on the community order and unity. This could be shown by increased interpersonal violence during crises and times of unrest like pandemics [16]. However, there are limited rigorous studies that estimated an increase in reporting of violence during or post-pandemic though media reports and anecdotal evidence are widespread [15].

Violence and its impact could be preventable in the same way public health efforts have prevented and reduced pregnancy-related complications, infectious diseases, and many other injuries all over the world. However, there are obstacles that hinder the system not to be effective, including a lack of accurate data on the magnitude and its determinants [17]. Therefore, given the alarming increase of natural disasters including pandemics during the recent decades, it is time to design and conduct more methodological sound studies [18, 19].

A preliminary search of PROSPERO, MEDLINE, the Cochrane Database of Systematic Reviews, and the *JBI Database of Systematic Reviews and Implementation Reports* was conducted, and no current or underway systematic reviews on the topic were identified.

## Methods

The development of this protocol reasonably followed the Preferred Reporting Item for Systematic Reviews and Meta-Analyses Extension for a Protocol (PRISMA-P) guidelines (Supplementary file 1).

### Review objective

The aim of this review is to synthesize the best available evidence on the prevalence of domestic violence during catastrophic disease outbreaks including the COVID-19 pandemic.

### Study design

The proposed systematic review will be conducted in accordance with the Joanna Briggs Institute methodology for systematic reviews of prevalence and incidence and the Preferred Reporting Items for Systematic Reviews and Meta-Analyses (PRISMA) [20, 21].

### Inclusion criteria

#### Participants

The review will consider studies that include children, adolescents, women, men, elders, and any vulnerable groups with or without co-existing health problems.

#### Condition

This review will consider studies that report on the prevalence of domestic violence in one or more forms: physical, psychological, and sexual during catastrophic health events (including pandemics and epidemics).

#### Context

This review will consider studies that report on the prevalence of domestic violence in the community which is conducted elsewhere in the globe.

### Types of studies

The review will consider observational studies and particularly population-based cross-sectional studies. Other study designs that provide indications of the prevalence of domestic violence during catastrophic health events will be considered for inclusion.

Studies published in the English language will be included, while we will not have a restriction to the sample size of the studies. Studies published after 2010 G.C. will be included in the review to capture recent trends in the prevalence of domestic violence during disease epidemics and pandemics.

### Search strategy

The search strategy will aim to locate both published and unpublished studies. An initial limited search of MEDLINE and CINAHL will be undertaken to identify articles on the topic. The text words contained in the titles and abstracts of relevant articles and the index terms used to describe the articles will be used to develop a full search strategy for the report of the name of the relevant database (Table 1). The search strategy, including all identified keywords and index terms, will be adapted for each included information source. Searching for additional studies from grey literature from government departments, international agencies, and academic institution repositories will be conducted using similar keywords from the search strings. The reference list of all studies selected for critical appraisal will be screened for additional studies.

**Table 1:** Search strategy of PubMed database

MEDLINE (PubMed). Date searched: 13 October 2020 Results retrieved: 8194 (((((((“epidemiology”[Subheading] OR “epidemiology”[All Fields] OR “prevalence”[All Fields] OR “prevalence”[MeSH Terms]) AND (“domestic violence”[MeSH Terms] OR (“domestic”[All Fields] AND “violence”[All Fields]) OR “domestic violence”[All Fields])) OR “violence”[MeSH Terms]) OR “physical abuse”[MeSH Terms]) AND “sex offenses”[MeSH Terms]) OR (“disease outbreaks”[MeSH Terms] OR (“disease”[All Fields] AND “outbreaks”[All Fields]) OR “disease outbreaks”[All Fields] OR (“disease”[All Fields] AND “outbreak”[All Fields]) OR “disease outbreak”[All Fields])) OR “epidemics”[MeSH Terms]) AND “pandemics”[MeSH Terms]	

### Information sources

The databases to be searched include PubMed, Scopus, Google Scholar, Embase, Web of Science, survey reports, conference papers, dissertations, bulletins, and fact sheets. The trial registers to be searched include Cochrane Central Register of Controlled Trials and the search for unpublished studies will include survey reports.

### Study selection

Following the search, all identified citations will be collated and uploaded into Mendeley v1.19.6 (Mendeley Ltd., Elsevier, the Netherlands) and duplicates removed. Titles and abstracts will then be screened by two independent reviewers for the assessment against the inclusion criteria for the review. Potentially relevant studies will be retrieved in full, and their citation details imported into the Joanna Briggs Institute System for the Unified Management, Assessment and Review of Information (JBI SUMARI) [22]. The full text of the selected citations will be assessed in detail against the inclusion criteria by two independent reviewers. Reasons for exclusion of full-text studies that do not meet the inclusion criteria will be recorded and reported in the systematic review. Any disagreements that arise between the reviewers at each stage of the study selection process will be resolved through discussion, or with a third reviewer. The results of the search will be reported in full in the final systematic review and presented in a Preferred Reporting Items for Systematic Reviews and Meta-analyses (PRISMA) flow diagram [23].

### Assessment of methodological quality

Eligible studies will be critically appraised by two independent reviewers for methodological quality using standardized critical appraisal instruments for prevalence studies in JBI SUMARI [20] (Table 2). Authors of the papers will be contacted to request missing or additional data for clarification, where required. Any disagreements that arise will be resolved through discussion or with a third reviewer. The results of the critical appraisal will be reported in narrative form and in a table.

**Table 2:** Critical appraisal tool for included studies

Study design	Tool	Domains	Overall risk of bias judgement
Cross-sectional studies	JBI Critical Appraisal Checklist for Studies Reporting Prevalence Data	1. Was the sample frame appropriate to address the target population? 2. Were study participants sampled in an appropriate way? 3. Was the sample size adequate? 4. Were the study subjects and the setting described in detail? 5. Was the data analysis conducted with sufficient coverage of the identified sample? 6. Were valid methods used for the identification of the condition? 7. Was the condition measure in a standard, reliable way for all participants? 8. Was there appropriate statistical analysis? 9. Was the response rate adequate, and if not, was the low response rate managed appropriately?	An item would be scored ‘0’ if it was answered ‘no’ or ‘unclear’; if it was answered ‘yes’, then the item scored ‘1’. Methodological quality will be considered ‘low’, ‘moderate’, and ‘high’ if three or less, four to six, and seven to nine criteria will be met, respectively.

### Data extraction

Data will be extracted from papers included in the review using the standardized data extraction tool for prevalence and incidence studies [24] by two independent reviewers. The data extracted will include specific details about the condition, populations, study methods, and proportions of interest to the review question and specific objectives including (Appendix):

i. General information: author/s name, study title and aim, year and country of study, publication type or source of data (journal or report), and socioeconomic data
ii. Study characteristics: study design, characteristics of the study population (demographic and socio-economic), sample size, and sampling methods
iii. Outcome information: proportion of people reported with either current or lifetime prevalence of domestic violence during diseases outbreaks

Any disagreements that arise between the reviewers will be resolved through discussion or with a third reviewer. The authors of the papers will be contacted to request missing or additional data where required.

### Data synthesis

Papers will, where possible, be pooled in a statistical meta-analysis using RevMan 5.3 (Copenhagen: The Nordic Cochrane Centre, Cochrane) [25] or any other relevant software. Effect sizes will be expressed as a proportion with 95% confidence intervals around the summary estimate. Heterogeneity will be assessed statistically using the standard chi-squared, tau-squared, and *I*-squared tests [26]. A random effects model using the double arcsine transformation approach will be used. Subgroup analyses will be conducted where there is sufficient data to investigate the disparities in the prevalence of the outcome of interest among different population groups (women, men, adolescents, elders, etc.), types of violence (physical, psychological, and sexual), and WHO regions (African Region (AFRO), the Eastern Mediterranean Region (EMRO), the South-East Asia Region (SEARO), the Region of the Americas (AMRO), the Western Pacific Region (WPRO), and the European Region (EURO)). Sensitivity analyses will be conducted to explore the impact of individual studies on the overall calculated prevalence estimates. This will be performed by investigating whether adding or dropping primary studies with slightly unidentified types of domestic violence will make a difference.

Where statistical pooling is not possible due to heterogeneity, the findings will be presented in a narrative form including tables and figures to aid in data presentation where appropriate. Source of heterogeneity and reason for which it is determined to be inappropriate to pool data will be specified in the systematic review report.

## Discussion

Nowadays, our world is facing disastrous events than before, and among these, disease outbreaks be it epidemic or pandemic are the major ones. These disease outbreaks have their own direct impact on the well-being of the population in general and more severe impact on the most vulnerable groups, specifically. Beyond a deteriorated health outcome due to the diseases, there is equal or most probably high negative impact on the lives of the poor which will be long lasting and irreversible. We have seen from our last experience that during specific disease epidemics, prevalence of domestic violence was almost twofold than the time when there was no epidemics. This systematic review will contribute to what is known, giving novel attention to the challenges and thus the need for intervention mechanisms to combat the challenge. This could be considered as a necessary step toward the prevention of domestic violence during catastrophic health events. Though we will not include non-English studies, which may cause a publication bias, we will comprehensively retrieve the reference lists of the studies included to obtain more additional studies. Hence, the synthesis can meet the proposed objectives and answers the research questions. The results of the review will inform policymakers, healthcare providers, and researchers to develop need-based and timely interventions aimed at preventing domestic violence during catastrophic health events.

### Supplementary Information

**Additional file 1.**

## Data Availability

Not applicable.

## Appendix

### Data extraction instrument

#### JBI data extraction form for prevalence and incidence studies

**Table:**

**JBI data extraction form for prevalence and incidence studies** Study details Reviewer – Study ID/Record Number - Date – Study title – Author – Year – Journal – Aims of the study – Study method Setting – Study design – Follow-up or study duration – Subject characteristics – Dependent variable – Outcomes – Outcome measurements – Ethical approval – Method of data analysis – Results Prevalence *n*/*N* (%) – Proportion and 95% confidence intervals – Incidence *n*/*N* (%) – Proportion and 95% confidence intervals and duration of recruitment or the study – Authors’ comments – Reviewer comments –	

## Abbreviations

COVID-19: Coronavirus disease-19
JBI SUMARI: Joanna Briggs Institute - the System for the Unified Management, Assessment and Review of Information
PRISMA: Preferred Reporting Items for Systematic Reviews and Meta-Analyses
WHO: World Health Organization
